# Abdominal Magnetic Resonance Imaging (MRI) Findings Prompting the Diagnosis of Paroxysmal Nocturnal Hemoglobinuria: A Case Report

**DOI:** 10.7759/cureus.116645

**Published:** 2026-09-21

**Authors:** Camila Velez, Ana Maria Ramirez, Sebastian Peláez, Sebastián Arredondo

**Affiliations:** 1 Department of General Medicine, Universidad Pontificia Bolivariana, Medellín, COL; 2 Department of General Medicine, Universidad CES, Medellín, COL; 3 Department of Radiology, Diagnóstico Cedimed, Medellín, COL; 4 Department of Radiology, Universidad Pontificia Bolivariana, Medellín, COL

**Keywords:** abdominal, budd-chiari syndrome, hemosiderosis, magnetic resonance imaging, paroxysmal nocturnal hemoglobinuria

## Abstract

Paroxysmal nocturnal hemoglobinuria (PNH) is a hematopoietic disorder with a nonspecific clinical presentation that often delays the diagnosis. We present the case of a 32-year-old female patient who presented with diffuse abdominal pain, fatigue, and anemia. An abdominal ultrasound was performed, revealing hepatosplenomegaly, and a subsequent abdominal magnetic resonance imaging (MRI) identified diffuse, symmetrical renal cortical hypointense signal, consistent with chronic iron deposits due to intravascular hemolysis, together with absent hepatic venous flow and lack of venous enhancement, indicating chronic hepatic venous outflow obstruction consistent with Budd-Chiari syndrome. The combination of renal hemosiderosis with venous thrombosis at an unusual site prompted a hematological evaluation, which confirmed the diagnosis. This case reinforces a well-recognized imaging pattern that helps radiologists suspect this entity, thereby enabling timely diagnosis and treatment.

## Introduction

Paroxysmal nocturnal hemoglobinuria (PNH) is an acquired clonal disorder of the hematopoietic stem cell, caused by a somatic mutation in the phosphatidylinositol glycan anchor biosynthesis class A (PIG-A) gene, which prevents the synthesis of glycosylphosphatidylinositol, a glycolipid that anchors proteins to the cell membrane, and results in the absence of complement regulatory proteins, such as CD55 and CD59 [1-3], making erythrocytes, granulocytes, and monocytes susceptible to the lytic activity of complement. This results in the classic triad of PNH: intravascular hemolysis, bone marrow failure, and a predisposition to thrombosis in atypical venous sites, most commonly in hepatic and abdominal veins [2].

PNH is a rare disorder, with an estimated incidence of 1.3 new cases per million people per year [2]. The International PNH Registry included 1,610 patients from 273 centers in 25 countries; the median age at disease onset was 32 years, and 53.2% of patients were women [4].

PNH often presents with nonspecific symptoms, which can delay diagnosis [5]. In the same registry, the most frequently reported symptoms were fatigue (80%), dyspnea (64%), headache (63%), and hemoglobinuria (62%); abdominal pain and dysphagia were also commonly reported [4]. Hemoglobinuria, contrary to the name of the disease, is most common in the morning [2]. During hemolytic crises, patients may experience malaise, fever, and abdominal or lower back pain, symptoms similar to those caused by vascular thrombosis [1].

Laboratory findings may reveal cytopenias affecting a single cell line, ranging from mild to severe, along with reticulocytosis and elevated lactate dehydrogenase levels [5]. A negative Coombs test helps distinguish this condition from autoimmune hemolytic anemias [5].

Flow cytometry, the test of choice, confirms PNH by demonstrating a deficiency of glycosylphosphatidylinositol-linked antigens on erythrocytes, granulocytes, and monocytes in peripheral blood [2,5]. However, this test is only ordered when there is prior suspicion, and the nonspecific clinical manifestations make it difficult to suspect [5].

In this setting, imaging may provide the first diagnostic clue. We present the case of a young woman in whom contrast-enhanced abdominal magnetic resonance imaging (MRI) demonstrated the combination of bilateral renal cortical hemosiderosis and chronic hepatic venous outflow obstruction. Although this combination has been described previously [1,6], it remains underrecognized in routine reporting, and in this patient, it was the finding that raised suspicion of PNH before hematologic confirmation.

## Case presentation

A 32-year-old female patient, a resident of Amalfi (rural area), Colombia, with no significant past medical history, presented with diffuse abdominal pain of six months' duration, associated with progressive fatigue and anemia. As part of the initial clinical workup, an abdominal ultrasound revealed hepatomegaly and splenomegaly. To further characterize these findings, a contrast-enhanced abdominal MRI study was performed during the same diagnostic workup on a 1.5-T Siemens system (Forchheim, Germany), including axial b=0 diffusion-weighted imaging (DWI), coronal T2-weighted half-Fourier acquisition single-shot turbo spin-echo (HASTE), axial T1-weighted gradient-echo in-phase and opposed-phase sequences, and delayed axial T1-weighted volumetric interpolated breath-hold examination (VIBE) sequences after the intravenous administration of gadobutrol (Gadovist).

MRI demonstrated diffuse, bilateral, and symmetric hypointensity of the renal cortex. The abnormal cortical signal was most conspicuous on T2-weighted HASTE images, with relative preservation of the renal medulla, resulting in an apparent increase in corticomedullary contrast (Figure 1). The cortical hypointensity was substantially less conspicuous on fat-suppressed T2-weighted images. On T1-weighted gradient-echo in-phase images, the renal cortex also demonstrated diffuse low signal intensity (Figure 1). This finding was markedly less conspicuous on opposed-phase imaging, consistent with a magnetic susceptibility effect. Differences in cortical signal conspicuity between in-phase, opposed-phase, and fat-suppressed acquisitions also depend on echo time and other sequence parameters and were therefore interpreted as supportive rather than confirmatory of iron deposition. On dynamic three-dimensional T1-weighted gradient-echo (VIBE) sequences, diffuse cortical hypointensity persisted throughout all contrast-enhanced phases, including arterial, portal venous, and equilibrium imaging (Figure 1). DWI and apparent diffusion coefficient (ADC) maps showed no significant renal abnormalities.

**Figure 1:**
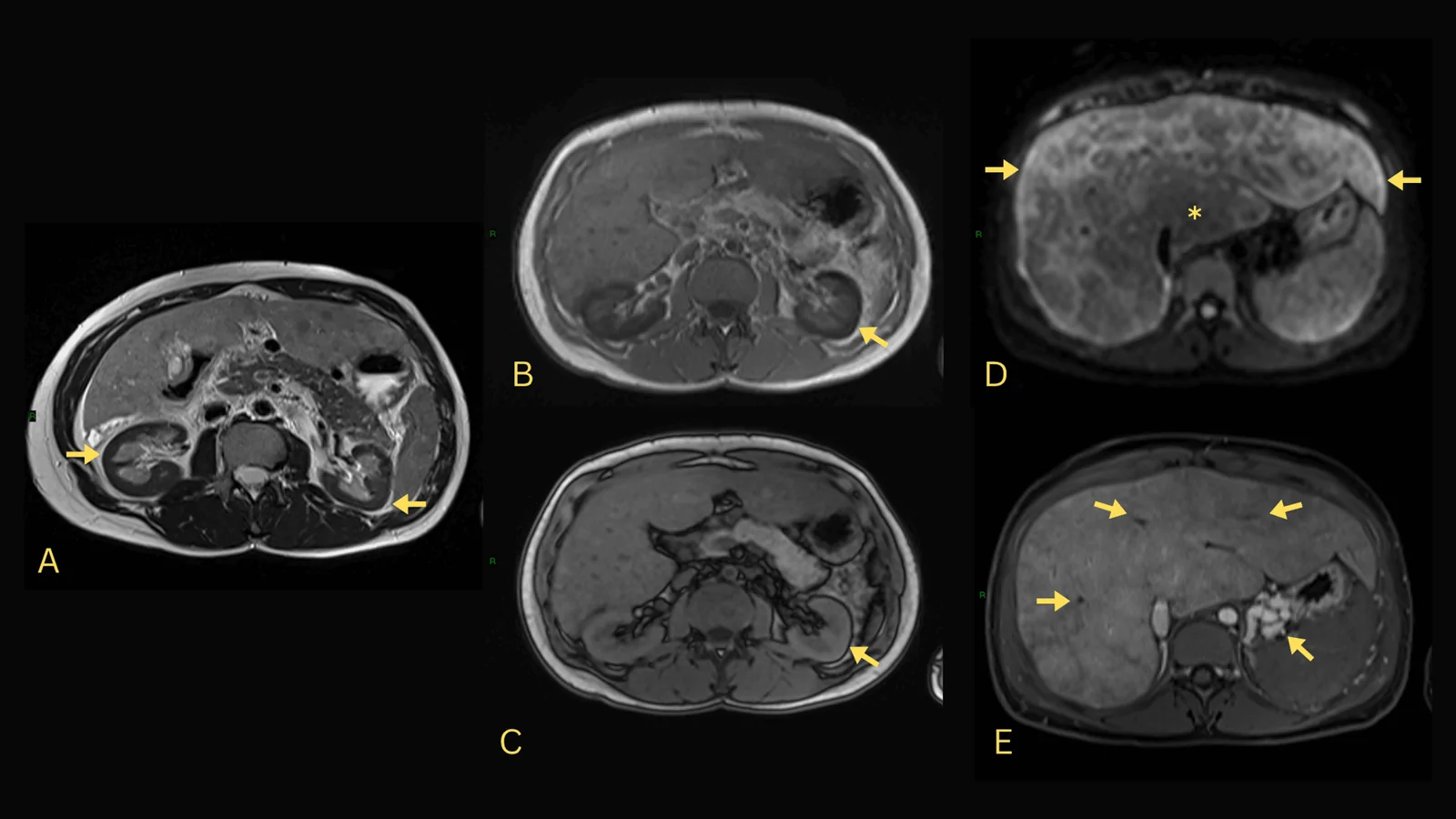
Contrast-enhanced abdominal magnetic resonance imaging A 32-year-old female patient with paroxysmal nocturnal hemoglobinuria. (A) An axial T2-weighted HASTE image demonstrates marked diffuse hypointensity of the bilateral renal cortex (arrows), consistent with abnormal renal iron deposition. (B) An axial T1-weighted gradient-echo in-phase image (TR 198 ms, TE 5.16 ms) demonstrates bilateral renal cortical hypointensity (arrow), consistent with abnormal renal iron deposition. (C) The corresponding axial T1-weighted gradient-echo opposed-phase image (TR 188 ms, TE 2.22 ms) shows the normalization of the bilateral renal cortical signal (arrow), further supporting abnormal iron deposition. (D) An axial b=0 diffusion-weighted image demonstrates hepatomegaly with subcapsular and periportal edema (arrows), with relative sparing of the central hepatic parenchyma, particularly the caudate lobe (asterisk). (E) An axial delayed T1-weighted VIBE gradient-echo image after intravenous gadobutrol administration demonstrates the absence of enhancement of the hepatic veins (arrows), with prominent perigastric venous collaterals (arrowhead). HASTE: half-Fourier acquisition single-shot turbo spin-echo; TR: repetition time; TE: echo time; VIBE: volumetric interpolated breath-hold examination

In addition to the renal findings, abdominal MRI demonstrated imaging features of chronic hepatic venous outflow obstruction. The hepatic parenchyma exhibited a heterogeneous peripheral signal abnormality, best appreciated on diffusion-weighted images with a b value of 0 s/mm² (Figure 1), consistent with chronic hepatic congestion. Relative preservation of the central hepatic parenchyma, particularly the caudate lobe, was observed, likely reflecting compensatory collateral venous drainage. Normal flow voids of the hepatic veins were absent on all pulse sequences. Following intravenous gadolinium administration, the hepatic veins failed to enhance during the portal venous, equilibrium, and delayed phases (Figure 1). Additionally, enhancing splenorenal and perigastric venous collaterals were identified (Figure 2). Taken together, these findings were consistent with chronic hepatic venous outflow obstruction. Associated morphologic features of chronic liver disease were also present.

**Figure 2:**
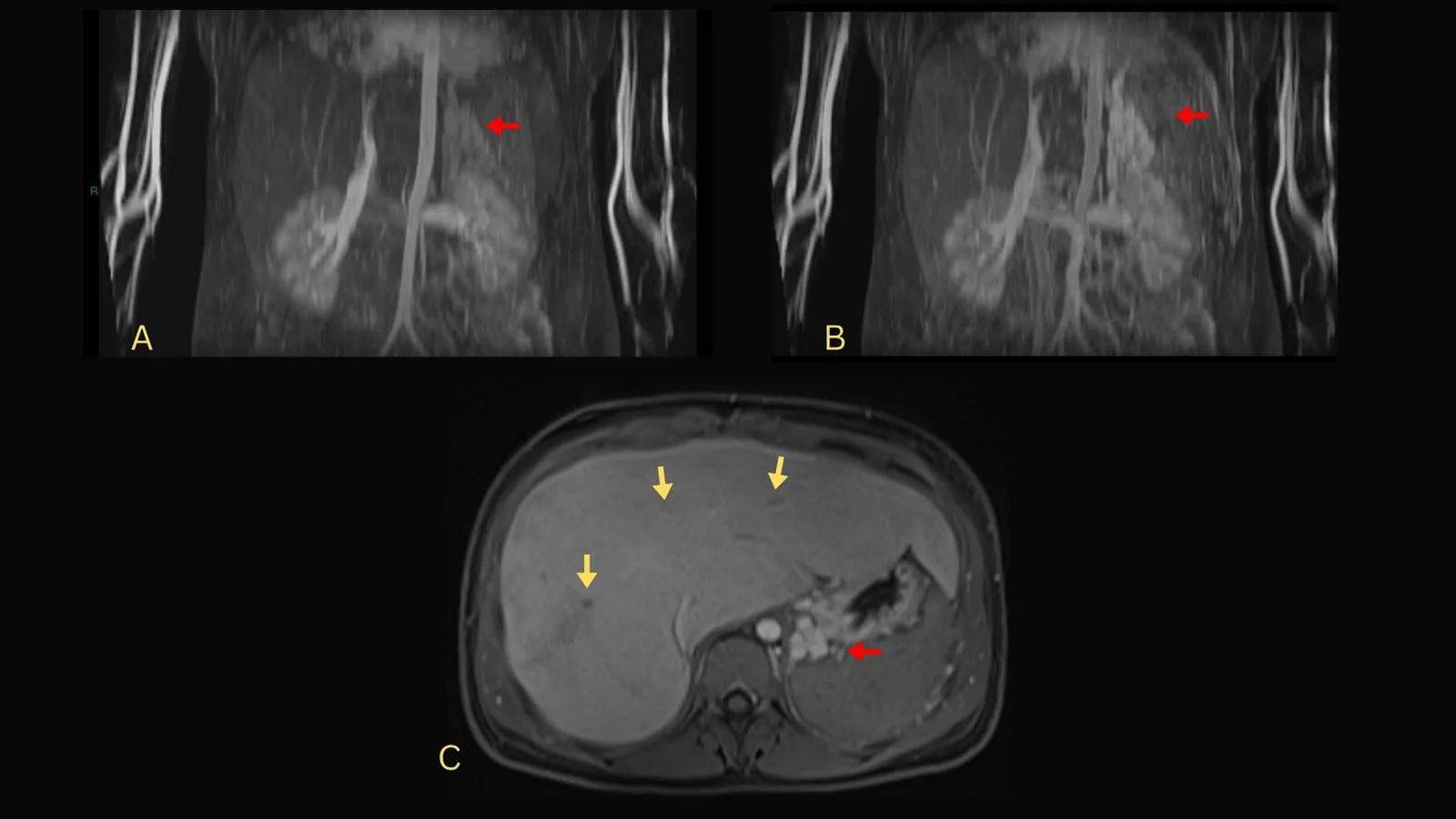
Vascular-phase and delayed post-contrast imaging of the hepatic veins and venous collaterals (A) Venous-phase MIP magnetic resonance venographic reconstruction; the red arrow indicates venous collaterals. (B) Late-phase MIP venographic reconstruction; the red arrow indicates venous collaterals. (C) Delayed post-contrast T1-weighted VIBE source image; the red arrow indicates venous collaterals, and the yellow arrows indicate no enhancement in the hepatic veins. MIP: maximum intensity projection; VIBE: volumetric interpolated breath-hold examination

The combination of bilateral diffuse renal cortical hemosiderosis and imaging findings consistent with chronic Budd-Chiari syndrome raised strong suspicion for PNH. Based on the imaging findings, the patient was referred for hematologic evaluation. Approximately six months after presentation, flow cytometry confirmed PNH, demonstrating clone populations of 32.5% in erythrocytes, 66% in monocytes, and 77% in neutrophils. Doppler ultrasonography performed as part of the same diagnostic evaluation reported filiform hepatic veins with monophasic flow and poor visualization of their drainage into the inferior vena cava, consistent with chronic hepatic venous outflow obstruction. One month later, lactate dehydrogenase (LDH) was elevated to 1,214 U/L (reference range: 200-480 U/L), supporting ongoing hemolytic activity. Eculizumab and anticoagulation were then initiated, and anticoagulation was suspended at the one-year follow-up visit. At five years of follow-up, the patient reported a notable improvement in her condition with resolution of clinical manifestations and no associated complications.

## Discussion

The most characteristic imaging finding of PNH is bilateral symmetric renal cortical hypointensity on MRI, which results from progressive hemosiderin deposition within the proximal renal tubular epithelial cells secondary to chronic intravascular hemolysis [1,4,6,7]. This imaging pattern reflects glomerular filtration of free hemoglobin followed by tubular reabsorption and intracellular iron accumulation. Hemosiderin accumulates specifically within the epithelium of the proximal convoluted tubules, which are confined to the renal cortex, and involves the entire cortex, including the columns of Bertin; the medulla, which does not participate in the reabsorption of filtered hemoglobin, retains normal signal intensity, producing the reversed corticomedullary differentiation [5,7]. On MRI, iron deposition produces a marked susceptibility effect, leading to diffuse loss of cortical signal intensity, particularly on iron-sensitive sequences such as T2- and T2*-weighted and gradient-echo images [6,7].

In our patient, diffuse bilateral renal cortical hypointensity, most conspicuous on T1-weighted gradient-echo and T2-weighted HASTE sequences, with characteristic relative sparing of the medulla, was consistent with chronic cortical hemosiderin deposition secondary to intravascular hemolysis [1,5,8]. Differences in cortical signal conspicuity between in-phase, opposed-phase, and fat-suppressed acquisitions likely reflect variations in echo time and other sequence parameters and should not independently be regarded as confirmation of iron deposition.

Although this pattern of renal cortical hemosiderosis is highly suggestive of PNH, it is not pathognomonic, since diffuse bilateral cortical hypointensity may also occur in other conditions associated with chronic intravascular hemolysis or systemic iron overload. The main differential diagnosis is mechanical hemolysis due to a prosthetic valve, which produces hyperattenuation on computed tomography (CT) and an MRI appearance identical to PNH, making the clinical findings and the patient's history the main distinguishing features [7,9]. Sickle cell disease may also show bilateral cortical hypointensity on MRI during crisis or in polytransfused patients [7,10]. Still, the main difference is that, on both CT and MRI, patients present with cortical scarring and infarcts due to microvascular disease [7,10]. Hemolytic uremic syndrome is considered a differential diagnosis for renal cortex involvement, but it has a different mechanism from iron deposition. The initial lesion shows thrombotic microangiopathy with no iron deposits in the parenchyma; in the acute phase, T2 and T2* signal intensities are preserved, with absent or inverted corticomedullary contrast on diffusion maps [11]. Renal cortical hypointensity appears only on conventional sequences if the disease progresses to cortical necrosis, due to calcification rather than hemosiderin [7]. Thalassemia is included in the differential diagnosis for the opposite reason: despite marked systemic iron overload, iron deposits are usually found in the liver, spleen, bone marrow, and pancreas [7]; the renal cortex is typically unaffected [12]; and a hypointense renal cortex requires intravascular hemolysis [7,9], not only systemic iron overload. This explains the MRI with normal cortical signal [9,12]. In our patient, the absence of significant abnormalities on DWI and ADC maps supported chronic iron deposition rather than an acute inflammatory or ischemic renal process.

The second key imaging finding was chronic hepatic venous outflow obstruction. Poor visualization and absence of definite contrast opacification of the hepatic veins, together with heterogeneous peripheral hepatic congestion, relative caudate-lobe preservation, and venous collateral formation, were consistent with Budd-Chiari syndrome [1,5,6,8]. Magnetic resonance venographic reconstructions further demonstrated poor opacification of the hepatic veins with enhancing splenorenal and perigastric collaterals (Figure 2). Because absent flow voids or non-opacification on MRI may also result from slow flow or technical factors, correlation with Doppler ultrasonography was performed; this showed filiform hepatic veins with monophasic flow and poor visualization of their drainage into the inferior vena cava, supporting chronic hepatic venous outflow obstruction rather than a flow-related or technical artifact. Thrombosis represents the leading cause of morbidity and mortality in PNH, occurring in up to 44% of patients during the course of the disease [5]. Unlike other inherited or acquired thrombophilic disorders, PNH has a marked predilection for thrombosis in unusual venous territories, particularly the hepatic veins, portal vein, mesenteric veins, and cerebral venous sinuses [2,5]. Therefore, the identification of Budd-Chiari syndrome in a young patient without an obvious predisposing condition should prompt the consideration of an underlying hematologic disorder.

The principal contribution of this case is to reinforce the diagnostic value of recognizing renal cortical hemosiderosis and Budd-Chiari syndrome as a combined imaging pattern of PNH. Although this association has previously been described [1,6], its identification on abdominal MRI may raise suspicion for PNH before hematologic confirmation. In our patient, abdominal MRI was performed to investigate chronic abdominal pain and unexplained anemia rather than to evaluate a suspected hematologic disease. It was the integration of the renal and hepatic imaging findings that suggested an underlying chronic intravascular hemolytic disorder and prompted flow cytometric analysis, which subsequently confirmed the diagnosis of PNH.

This observation has important implications for radiologic practice. Renal cortical hypointensity and hepatic venous thrombosis are often described as independent findings without recognition of their underlying pathophysiologic relationship. Our case illustrates that radiologists can play a much more active role in the diagnostic process by recognizing this characteristic imaging pattern. Rather than merely reporting isolated abnormalities, integrating these findings may suggest a specific underlying diagnosis, expedite hematologic evaluation, and facilitate the early initiation of complement inhibitor therapy before potentially life-threatening thrombotic complications occur.

Early recognition matters because effective therapy is available. The management of PNH depends on disease severity and the presence or absence of thromboembolic complications. Complement inhibitors such as eculizumab have become the primary pharmacological strategy, significantly reducing intravascular hemolysis and thromboembolic rates compared with untreated patients [3,13]. Once Budd-Chiari syndrome has developed, treatment should include anticoagulants and diuretics as a first-line therapy [14]. Transjugular intrahepatic portosystemic shunt (TIPS) can be performed safely even in the presence of PNH, with a reported success rate of 86%, comparable to the 82% success rate observed in patients with Budd-Chiari syndrome without PNH according to the EN-Vie cohort [3,14]. Hematopoietic stem cell transplant remains the only curative treatment for PNH, and it has been used successfully to manage both the hematologic disease and its thrombotic complications, with five out of six transplant recipients achieving cure [14]. The overall prognosis has improved substantially in recent decades: in the same cohort, the two-year survival rate among patients with Budd-Chiari syndrome and underlying PNH was 93%, with no significant differences in survival compared with patients with Budd-Chiari syndrome without PNH, representing a marked improvement over historical mortality estimates, which reached as high as 67% in previous case series [14].

In our patient, recognition of this imaging pattern directed the diagnostic workup toward an uncommon hematologic disorder, which was subsequently confirmed by flow cytometry. Treatment with eculizumab and anticoagulation resulted in a favorable clinical course without recurrent thrombotic events during five years of follow-up, highlighting the potential clinical impact of the accurate interpretation of abdominal MRI findings.

## Conclusions

The combination of bilateral renal cortical hemosiderosis and chronic hepatic venous outflow obstruction (Budd-Chiari syndrome) is a previously described, highly suggestive imaging pattern of PNH. Although neither finding is pathognomonic in isolation, identifying both on abdominal MRI may precede definitive hematologic diagnosis and provide the first opportunity to suspect this rare disorder.

This case reinforces that abdominal MRI should not be regarded solely as a tool for assessing PNH complications but also as a valuable modality that can suggest the diagnosis when radiologists integrate renal and hepatic vascular findings within the same pathophysiologic context. Radiologists who encounter this pattern should look beyond the isolated description of cortical signal loss and hepatic vein thrombosis (or venous thrombosis at other unusual sites); instead, they should correlate these findings to raise syndromic suspicion and prompt targeted hematologic investigation. Recognition of this imaging pattern may accelerate confirmation by flow cytometry, facilitate the timely initiation of targeted therapy, and ultimately improve the prognosis of a rare disease associated with substantial thrombotic morbidity and mortality.
